# Online Availability and Safety of Drugs in Shortage: A Descriptive Study of Internet Vendor Characteristics

**DOI:** 10.2196/jmir.1999

**Published:** 2012-02-09

**Authors:** Bryan A Liang, Tim K Mackey

**Affiliations:** ^1^Institute of Health Law StudiesCalifornia Western School of LawSan Diego, CAUnited States; ^2^San Diego Center for Patient SafetyDepartment of AnesthesiologyUniversity of California, San Diego - School of MedicineSan Diego, CAUnited States; ^3^Joint Doctoral Program in Global HealthUniversity of California, San Diego - San Diego State UniversitySan Diego, CAUnited States

**Keywords:** Illicit online pharmacies, FDA drug shortage, gray market, drug supply, social media, direct-to-consumer advertising, Internet pharmacies, health policy

## Abstract

**Background:**

Unprecedented drug shortages announced by the US Food and Drug Administration (FDA) have severely affected therapeutic access, patient safety, and public health. With continued shortages, patients may seek drugs online.

**Objective:**

To assess the prevalence of online marketing for current FDA shortage drugs and potential patient safety risks.

**Methods:**

We performed a descriptive study of the prevalence of online marketing for shortage drugs—that is, offers for sale of each drug, including characteristics of online drug sellers and intermediary sites marketing these drugs.

**Results:**

Of the 72 FDA shortage-listed drugs, 68 (94%) were offered for sale online. We found 291 offers for these drugs, the vast majority (n = 207, 71.1%) by online drug sellers selling direct to consumers. Intermediary sites included data aggregators (n = 22, 8%), forum links (n = 23, 8%), and personal page data links (n = 34, 12%), as well as Flickr social media links (n = 5, 2%), all advertising drugs without a prescription. Of the 91 online drug sellers identified, 31 (34%) had more than 1 shortage drug offered for sale, representing most (n = 148, 71%) of all online drug seller sales offers. The majority of these online drug sellers (n = 21, 68%) were on the National Association of Boards of Pharmacy (NABP) Not Recommended Sites list. Finally, for shortage drugs with an online drug seller (n = 58, 85%), 53 (91%) had at least one site on the Not Recommended list and 21 (36%) had only sites on the Not Recommended list.

**Conclusions:**

FDA shortage drugs are widely marketed over the Internet. Suspect online drug sellers and intermediaries dominate these sales offers. As a critical risk management issue, patients, providers, and policymakers should be extremely cautious in procuring shortage drugs through Internet sourcing.

## Introduction

The US Food and Drug Administration (FDA), the American Hospital Association, and the American Society of Health-System Pharmacists have announced a growing crisis of drug shortages in critical areas such as cancer therapy, urgent care, infectious disease, hypertension, orphan disease, pediatric, and other key patient and disease treatment categories [1-6]. According to the Premier healthcare alliance, over the last 6 months of 2010, 89% of providers experienced shortages that may have caused a medication safety issue or error in patient care, 80% experienced shortages that resulted in a delay or cancellation of a patient care intervention, and 98% experienced shortages that resulted in increased costs [7]. A survey by the American Hospital Association found similarly dramatic results, including 99.5% of surveyed hospitals reporting a drug shortage in the prior 6 months and 41% reporting shortages of more than 21 medicines during the same time frame [3].

This crisis has hit a critical juncture, with shortages leading to loss of patient lives and gross overpricing. The US Institute of Safe Medication Practices has estimated that 15 patients have died in the span of a mere 15 months because of these drug shortages [8,9]. Beyond patient mortality, hospitals have also been forced to delay treatment or leave patients untreated, in unnecessary pain, or with suboptimal clinical care, leading to complications, adverse events, and increased overall burden and costs for the health care system [3,7].

 The dearth of access to these key drugs has resulted in their being sought outside the traditional drug supply chain at virtually any price and condition. Indeed, Institute of Safe Medication Practices notes that hospitals have turned to the risky secondary or “gray” market (secondary resellers outside of direct drug distribution channels) in an effort to address the drug supply concern [10]. Those entering into these markets have faced markups as high as 4500%, with *average* markups of 650% and virtually all drugs at least double the normal market price [9,10]. Despite knowledge of risks to drug safety, including counterfeiting, diversion, and substandard and falsified drugs in this market, 12% of hospitals admitted to engaging in these purchases [8]. Documented fakes entering through the gray market include counterfeit cancer, human immunodeficiency virus, and diabetes drugs [8,11,12].

 However, of greatest concern is that, due to publicized profiteering, documented safety concerns, and diminishing availability of drugs in the gray market, patients affected by ongoing shortages may turn to a rapidly growing source: the Internet. However, the sale of drugs by illicit online pharmacies is of great concern, as they are a conduit for questionable therapeutic products for a wide range of treatments and diseases [13].

Indeed, purchasing drugs online is highly risky [14]. There is a significant criminal element in online drug sales, and the National Association of Boards of Pharmacy (NABP) has reported in its analysis that virtually all (96%) online drug sellers violate safe practices and/or pharmacy laws [15]. Further, there have been documented deaths from drugs obtained online, and the FDA, major national drug regulatory agencies, law enforcement, and the World Health Organization all warn against purchasing pharmaceuticals over the Internet [16-18].

Yet the challenges of drug supply shortages and exorbitant costs may drive patients in great need to seek drugs online. Given this potential crisis, we examined the availability of drugs subject to shortages on the Internet. We investigated online vendors of identified FDA current drugs in shortage to determine the characteristics of these sellers. Specifically, we examined the prevalence of online drug seller sites and intermediary sites including those sites that sell shortage drugs direct to patients, sites that act as intermediaries for the purchase of drugs and market online drug seller sites, and user-generated content via forums and social media that link to online drug sellers. We also determined whether sellers were accredited by the NABP’s Verified Internet Pharmacy Practice Sites (VIPPS) accreditation program, the only accredited online pharmacies recommended by the FDA [19]. This included determination of whether vendors were on the NABP Not Recommended Sites list. Not Recommended sites are Internet drug sellers that appear out of compliance with state and federal laws or NABP patient safety and pharmacy practice standards [20]. Finally, we also assessed characteristics that could have an impact on availability or legitimacy of product, such as “international” status, which have been identified as a suspect characteristic [21]. We did not compare prices or evaluate purchasing requirements, including validating whether sellers required prescriptions, because of ethical and legal concerns arising from fictitious claims of being a patient with a particular disease, and because recent work found that online drug sellers that purportedly “require” prescriptions in fact do not require them, use “surveys” in lieu of prescriptions, and/or illicitly sell prescriptions to purchasers [14,22]. Hence, the potential quality and safety of identified sites can be better determined by examining VIPPS accreditation or Not Recommended status as outlined above. These characteristics are also important in the context of the limited ability of consumers to identify suspect drug-selling websites [23].

We also did not assess location of website registration or purported sourcing (other than as international or domestic), as these have been found to be forensically difficult to rely upon for assessment of risk characteristics [14]. For example, websites listed in Canada have been found forwarding drug orders to Israel and then financial information to Russia, where the credit card transaction was processed, and the pharmaceutical was shipped from India to a consumer in the United States. Another operated a drug-selling scheme that had its domain name hosted in Korea and registered in St Kitts, with orders dispatched from Oklahoma City [14,24,25]. Other even more dramatic examples include a seller with a Web address in Russia, server in China, payee for the credit card charge in the United Kingdom, payment processing in Australia, and product mailed from Chicago, using a return address of an unsuspecting customer of the drug website; and an Internet drug seller in Costa Rica, with computer servers located in Cyprus, credit card payments processed in Israel, and revenues placed back in bank accounts in Cyprus [26]. As well, many online drug seller websites are mirror or affiliate sites—that is, duplicate selling pages that do not sell the product itself, but instead provide the main website with greater Web presence, with the mirror or affiliate site obtaining a percentage of sales [14]. There is no requirement generally for mirror sites to be located in the same geographic area as the main website.

## Methods

We first identified the FDA current shortages list of drugs with an endpoint of September 23, 2011, which included 72 drugs (*s* *hortage d* *rugs*) [27]. For each identified shortage drug, we searched Google using the search term *buy [* *drug* *]* where *drug* was the name of the specific shortage drug. Standard security settings were used in the browser, and no user was signed into the browser settings. We then identified the first 5 offers for sale or, if there were fewer than 5 sales offers, the number of sales offers within the first 5 search result pages. We used 5 sales offers as our number of hits to examine based on previous research that found that consumers purchasing goods online visit 3–5 websites prior to purchase [28,29]. For this analysis, we excluded wholesalers and sponsored links. Searches were performed from October 1, 2011 to October 17, 2011.

Internet sales offers were characterized in two categories. The first identified websites as *o* *nline d* *rug s* *eller* *s* (direct vendors of the shortage drug selling direct to consumers). Online drug sellers are sometimes called Internet pharmacies or online pharmacies, but since many if not most are in fact not pharmacies [15,20], we have not adopted this term. The second category identified *i* *ntermediary s* *ites* (websites that did not engage in the direct sale of drugs online, but acted as intermediaries or sources of information to purchase shortage drugs online). These included data aggregators (sites collecting data links for a particular shortage drug that links to online drug sellers); forum links (Internet discussion forums with links to online drug sellers); personal page data links (PPDLs, pages of personally placed links, materials, and other information that links to online drug sellers); and other social media links (eg, Facebook or Twitter).

For each identified online drug seller selling a shortage drug, we assessed whether the site was accredited by the NABP VIPPS program through review of the NABP VIPPS program website. We also assessed whether the online pharmacy was on the NABP Not Recommended Sites list, similarly reviewing the NABP Not Recommended Sites list on the NABP website [20]. We further assessed whether these online pharmacies were internationally or domestically based, regardless of their actual presence or ultimate location, as “international” status is a widely recognized characteristic of potential safety and quality concerns [21].

Finally, for intermediary sites, we assessed whether links advertised “no-prescription” drugs, an inherent indication of patient and drug safety risk as well as a violation of US law. Note that we did not include the term *no prescription* when searching for shortage drug advertisers. Non-VIPPS-accredited, international online drug sellers and intermediary sites linking to sites with no-prescription sales were deemed suspect sites.

## Results

We identified 72 shortage drugs (Table 1). When searched, 68 (94%) of these drugs were being advertised online. For these 68 shortage drugs, 291 total offers for sale resulted from online searches, the vast majority (n = 207, 71.1%) by online drug sellers selling direct to consumers. Intermediary sites advertising sales of drugs in shortage included data aggregator sources (n = 22, 8%), forum links (n = 23, 8%), and PPDLs (n = 34, 12%). We also identified Flickr social media links (n = 5, 2%). All intermediary sites and Flickr social media links sales offers (n = 84, 29%) advertised drug sales without a prescription. In total, there were 91 individually identified online drug sellers, 17 forum link sources, 11 Data Aggregators, 16 PPDL sources, and 5 Flickr links. These were coded using alphanumeric designators and are listed in Table 1.

**Figure 1:**
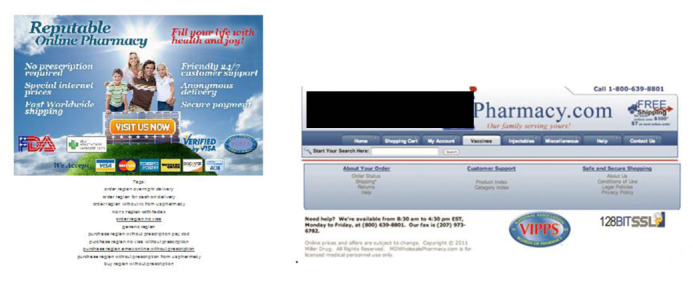
Unauthorized uses of Verified Internet Pharmacy Practice Sites (VIPPS) seal.

Most (n = 53, 58%) of the online drug sellers appeared to be of international origin, with Canada being the top purported source of these vendors (n = 32, 60%). We also found one online drug seller (online drug seller CB, Table 2) using the VIPPS accreditation seal, but the seller was not found on the VIPPS accreditation list. We also found one forum link also using an unauthorized VIPPS seal (see Figure 1).

Several online drug sellers dominated offers for shortage drugs (Table 2). Of the 91 vendors, 31 (34%) offered multiple (>1) drugs in shortage for sale, and were overrepresented with 148 (71%) of all online drug seller marketing events. Of these 31 sellers, 21 (68%) were on the NABP Not Recommended Sites list; 8 (26%) had no information available; only 1 (3%) was VIPPS accredited.

**Figure 2:**
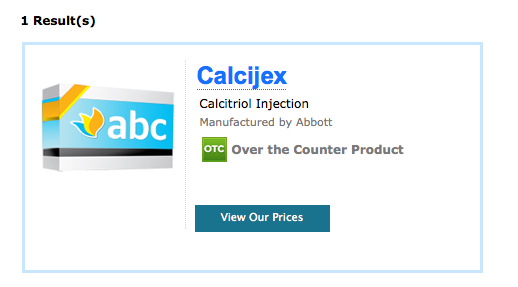
Shortage drug calcitriol injection advertised as an over-the-counter drug.

Finally, of the 68 drugs that were being offered for sale online, 58 (85%) were advertised for sale by at least one online drug seller (Table 1). Among these 58 drugs with online drug sellers, 53 (91%) were offered for sale by at least one Not Recommended NABP site. Indeed, 21 (36%) of these shortage drugs were advertised for sale *only* by NABP Not Recommended sites (Table 3). Only 4 drugs (7%) had any sales offers from a VIPPS-accredited pharmacy. We also found one shortage drug being advertised as over-the-counter when it is not (calcitriol; Figure 2). We also found other nonshortage drugs to be advertised as over-the-counter when they were not (eg, insulin or vaccines), as well as medical devices (eg, intrauterine devices).

**Table 1:** US Food and Drug Administration Current Drug Shortages list and Internet sellers (September 23, 2011)

Name of drug	Sellers
Acetylcysteine Inhalation Solution	Online drug sellers A^a^, B^a^, C^a^, D, E
Alcohol Dehydrated (Ethanol >98%)	Online drug sellers F, G, H
Amikacin Injection	Online drug sellers I, J^a^, L; forum link A
Amino Acid Injection	Data aggregator 1; forum links B, C
Aminocaproic Acid	Online drug sellers M^a^, N^a^, O^a^, P^a^
Ammonium Chloride Injection	Online drug seller Q^a^
Ammonium molybdate injection	No sales offers
Ammonul Injection 10%/10%	Online drug seller Q^a^; forum link D; PPDLs^b^ 1 (2×), 4
Amphetamine Mixed Salts, ER Capsules	Online drug sellers R, CL, S, T; PPDL 2
Anadrol-50 tablets (Oxymetholone Tablets)	Online drug sellers U, V, W^a^, X, Y
Aquasol A, 50,000 units/mL, 2 mL ampule	PPDL 1; online drug seller Q^a^; forum link E
Avalide	Online drug sellers Z^a^, AA, AB, A^a^, AD
Bleomycin Injection	Online drug sellers AE, AI; forum links F, H, I
Buprenorphine injection	PPDLs 1, 16; online drug seller AG^a^; data aggregators 2, 3
Calcitriol 1 mcg/mL Injection	Flickr 1; PPDL 3 (2×); online drug sellers Q^a^, C^a^
Calcium Chloride Injection	No sales offers
Calcium Gluconate 100 mg/mL	Online drug sellers AH^c^, AI, AL, C^a^
Cerezyme (imiglucerase for injection)	Online drug seller C^a^, Q^a^, AJ^a^; data aggregators 4, 5
Cisplatin injection 1 mg/mL solution	Online drug sellers L^a^, AK, AE; PPDL 4; data aggregator 5
Cyanocobalamin injection	Online drug sellers A^a^, AM, E, AN^a^, AO^a^
Cytarabine Injection	Online drug sellers AP^a^, AC^a^, AQ^a^, AR^a^, AS^a^
Daunorubicin hydrochloride solution for injection	Online drug sellers CK^a^, Q^a^, J^a^, K; data aggregator 6
Desmopressin Injection	Online drug sellers A^a^, AT^a^, AU^a^, AE; data aggregator 5
Dexamethasone Injection	Online drug sellers AV, J^a^, K, AW; PPDL 5
Digoxin Injection	Online drug sellers AJ^a^, AX; PPDLs 1, 6; data aggregator 7
Diltiazem Injection	PPDLs 1, 7; online drug sellers AY^a^, AK^a^, AZ^a^
Doxorubicin (adriamycin) lyophilized powder	Online drug sellers A^a^, BA, BB^a^, AH^c^
Doxorubicin Liposomal (Doxil) Injection	PPDL 1 (2×); online drug sellers Q^a^, AG; data aggregator 8
Doxorubicin Solution for Injection	Online drug seller BC, BD, BE^a^, L
Ethiodol (ETHIODIZED OIL) ampules	Online drug sellers Q^a^, BF; forum link F; PPDLs 4, 8
Etoposide solution for injection	Online drug sellers C^a^, A^a^; PPDL 9; forum link A
Fabrazyme (agalsidase beta)	Online drug sellers Q^a^, AJ^a^; data aggregator 4; PPDL 1
Fluorouracil Injection	Online drug sellers AS, L, AY^a^, AZ^a^, BH

Foscarnet Sodium Injection	Online drug sellers C^a^, Q^a^; data aggregators 4, 6; forum link G
Fosphenytoin Sodium Injection	Online drug sellers AS, Q^a^, AY^a^, AZ^a^; data aggregator 4
Furosemide Injection	Online drug sellers AP^a^, AJ^a^; PPDL 9; data aggregator 9; Flickr 2
Haloperidol Decanoate Injection	Online drug sellers BI^a^, AH^c^; Flickr 3; PPDL 1
Intravenous Fat Emulsion	Online drug sellers Q^a^; data aggregators 6, 5; PPDL 1; forum link H
Isoniazid Tablets	Online drug sellers AO^a^, BJ^a^, D; forum links I, A
Leucovorin Calcium Lyophilized Powder for Injection	Online drug sellers C^a^, AG^a^, AJ^a^, K; forum link J
Leuprolide Injection	Online drug sellers AT^a^, A^a^, BJ^a^, P^a^, AU^a^
Levoleucovorin (Fusilev) 50 mg single use vials	Online drug sellers Q^a^, BG; PPDL 1
Lorazepam Injection	Online drug seller BK
Magnesium Sulfate Injection	Online drug sellers BL^a^, J^a^, K, L, AY^a^
Methylphenidate HCl	Online drug sellers BM^a^, AG^a^, BN; forum links K, L
Metoclopramide injection	Online drug seller AJ^a^; forum links M, N, O; Flickr 4
Mitomycin Powder for Injection	Online drug sellers BO^a^, BP^a^, BQ, BE^a^, BR
Multi-Vitamin Infusion (Adult and pediatric)	PPDL 11; data aggregator 5
Nalbuphine Injection	Online drug sellers BS, BM^a^, BT; forum link P; PPDL 12
NeoProfen (ibuprofen lysine) Injection	Online drug sellers AJ^a^, AX^a^, BU; PPDL 13; forum link P
Neostigmine methylsulfate injection	Online drug sellers J^a^, AG^a^, K, AT; data aggregator 10
Neupro (rotigotine transdermal system)	Online drug sellers A^a^, Q^a^, AT^a^, AJ^a^, AO^a^
Norepinephrine Bitartrate Injection	Online drug sellers C^a^, Q^a^; data aggregator 6; forum links G, H
Ontak injection	Online drug seller Q^a^; data aggregators 4, 5; forum link G; PPDL 1
Oxsoralen (methoxsalen) 1% topical lotion	Online drug sellers AG^a^, AO^a^; data aggregator 10; forum link Q
Oxsoralen-Ultra (methoxsalen) 10 mg capsules	Online drug sellers Q^a^, AX^a^, BV, BW, AO^a^
Paclitaxel Injection	Online drug sellers BI^a^, BX, L, BY, D
Phenylephrine HCl Injection	PPDLs 14, 15
Potassium Phosphate Injection	No sales offers
Procainamide HCL Injection	Online drug sellers BZ, CA^a^, A^a^
Propofol Injection	Online drug sellers A^a^, C^a^, AT^a^, Q^a^, BB^a^
Sodium Chloride 23.4%	Online drug sellers Q^a^, AI, CC, CD, CB^d^
Sodium Chloride 14.6% Injection	Online drug seller AH^c^
Sodium Phosphate Injection	No sales offers
Streptomycin for Injection, USP	Online drug sellers CF^a^, BE^a^, Q^a^, CE
Sulfamethoxazole 80mg/trimethoprim 16mg/ml injection (SMX/TMP)	Online drug sellers Q^a^, AJ^a^, AX^a^, CG; data aggregator 11
Thiotepa for Injection	Data aggregators 4, 5

Thyrogen (thyrotropin alfa) injection 1.1mg/vial	Online drug sellers AJ^a^, CH^a^, Q^a^; data aggregator 4; PPDL 4
Thyrolar Tablets	Data aggregator 4
Vasopressin Injection	Online drug sellers J^a^, AS^a^, K, CI, CJ^a^
Vecuronium Injection	Online drug sellers Q^a^, AS, CM, AY; forum link G
Vincristine Sulfate Injection	Online drug sellers A^a^, AS, CM, L; PPDL 1

^a^ On National Association of Boards of Pharmacy (NAPB) Not Recommended Sites list for online drug purchasing.

^b^ Personal page data link.

^c^ NAPB Verified Internet Pharmacy Practice Sites (VIPPS)-accredited site.

^d^ Claims NABP VIPPS accreditation but not on NABP VIPPS accreditation list.

**Table 2:** Characteristics of Internet shortage drug sellers

Online drug seller	Marketing event frequency	NABP^a^ status	International/domestic (US) status
Q	23	Not recommended	International
A	12	Not recommended	International^b^
AJ	10	Not recommended	International^b^
C	9	Not recommended	International
K	7	No information	
L	7	No information	International
J	6	Not recommended	International^b^
AG	6	Not recommended	
AS	6	Not recommended	
AO	5	Not recommended	International^b^
AT	5	Not recommended	International^b^
AY	5	Not recommended	International^b^
AH	4	VIPPS^c^ accredited	US
AX	4	Not recommended	
AE	3	No information	International^b^
BJ	3	Not recommended	International^b^
D	3	No information	International^b^
AZ	3	Not recommended	International^b^
BE	3	Not recommended	International
P	2	Not recommended	International^b^
AI	2	No information	
AK	2	Not recommended	International^b^
AP	2	Not recommended	International
AU	2	Not recommended	International
BB	2	Not recommended	International^b^
BI	2	Not recommended	International^b^
BM	2	Not recommended	International^b^
CM	2	No information	International^b^
E	2	No information	International^b^
AP	2	Not recommended	
BG	2	No information	
N	1	Not recommended	International^b^
M	1	Not recommended	International^b^
B	1	Not recommended	International^b^
F	1	No information	
G	1	No information	
H	1	No information	
I	1	No information	International
O	1	Not recommended	International
R	1	No information	
S	1	No information	International
T	1	No information	
U	1	No information	
V	1	No information	
W	1	Not recommended	
X	1	No information	
Y	1	No information	International
AA	1	No information	
AB	1	No information	International
AD	1	No information	
AF	1	No information	
AL	1	No information	
AM	1	Not recommended	
AN	1	Not recommended	International^b^
AQ	1	Not recommended	International
AR	1	Not recommended	
AV	1	No information	
AW	1	No information	International^b^
BA	1	No information	International
BC	1	No information	International
BD	1	No information	International
BF	1	No information	
BH	1	VIPPS accredited	
BK	1	No information	
BL	1	Not recommended	International
BN	1	No information	International
BO	1	Not recommended	
BP	1	Not recommended	International^b^
BQ	1	No information	International^b^
BR	1	No information	International
BS	1	No information	
BT	1	No information	
BU	1	No information	International
BV	1	No information	International
BW	1	No information	International^b^
BX	1	No information	
BY	1	No information	International
BZ	1	No information	
CA	1	No information	International^b^
CB^d^	1	Not recommended	
CC	1	No information	
CD	1	No information	
CE	1	No information	
CF	1	Not recommended	International^b^
CG	1	No information	International^b^
CH	1	Not recommended	International^b^
CI	1	No information	
CJ	1	Not recommended	International^b^
CK	1	Not recommended	International
CL	1	No information	
Z	1	Not recommended	International^b^

^a^ National Association of Boards of Pharmacy.

^b^ Purported Canadian websites.

^c^ Verified Internet Pharmacy Practice Sites.

^d^ Use of VIPPS seal by nonaccredited vendor.

**Table 3:** US Food and Drug Administration Current Drug Shortage list drugs found only on National Association of Boards of Pharmacy’s Not Recommended online drug seller sites

Drug
Aminocaproic acid
Ammonul
Calcitriol
Cerezyme
Cytarabine
Diltiazem
Doxorubicin
Etoposide solution
Fabrazyme
Foscarnet
Furosemide
Intravenous fat emulsion
Leuprolide
Magnesium sulfate injection
Metoclopramide
Neupro
Norepinephrine injection
Ontak injection
Oxsoralen 1% topical
Propofol
Thyrogen injection

## Discussion

Patients face considerable risks when attempting to procure FDA current shortage drugs online. We find that suspect vendors populate much of the online market for these drugs, and international online drug sellers identified by NABP as Not Recommended dominate this eHealth landscape. With more than 90% of these drugs being offered for sale by at least one NABP Not Recommended site, in effect if an online drug seller is selling an FDA shortage drug, there is virtually always an NABP Not Recommended site selling in that market. Indeed, with more than a third of all of these drugs, including cancer, emergency, orphan drug, and other lifesaving therapeutic products, being sold by *only* NABP Not Recommended site vendors, it is highly likely that anyone searching online for drugs in shortage will encounter a suspect seller. Even those patients who may seek to legally procure a shortage drug online with a valid prescription may have a difficult time in identifying a legitimate online drug seller source, given oversaturation of suspect providers and marketing sources. This is of great patient safety concern, particularly in the context that most online drug sellers are suspect and research indicating that online drug sellers magnify positive aspects of online purchases but minimize discussion of associated risks [30].

**Figure 3:**
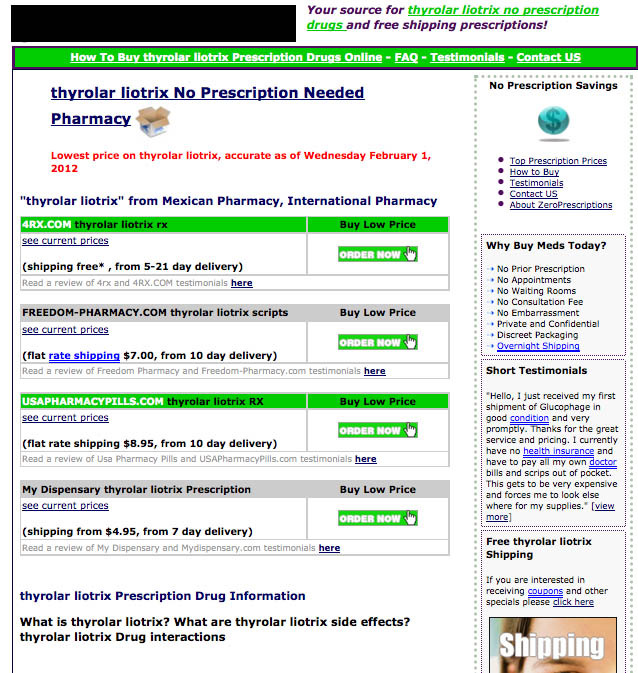
Intermediary site marketing products as no-prescription drug.

**Figure 4:**
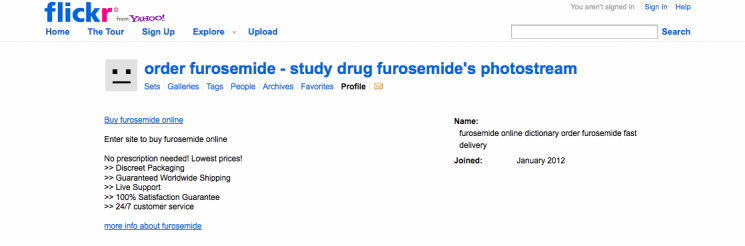
Flickr social media link for no-prescription shortage drug.

In addition, with 100% of the intermediary sites marketing these scarce medications as no-prescription drugs (see Figure 3), including social media sites such as Flickr that had been previously unobserved (see Figure 4) [29], this further indicates that the online marketplace for these treatments is extremely risky and diversifying. 

 These findings, in combination with previous work showing the extensive presence of illicit online drug sellers in other social media such as Facebook and Twitter [31,32], should place all stakeholders on alert about the legitimacy of online drug sellers, other vendors, and the quality of their products.

It is particularly troubling that so few NABP VIPPS-accredited pharmacies appeared in the returns from a common Google search. Even if legitimate pharmacies are marketing shortage drugs, the overwhelming presence of NABP Not Recommended online drug sellers may crowd out their arising early or at all in search results. The tremendous dominance of suspect online drug sellers and the virtual absence of authorized VIPPS-accredited online pharmacies for drugs in short supply may have an immediate impact on patient safety. This is particularly true in the context of other work reporting similar findings for online availability of biologics in short supply, which also include absence of VIPPS-accredited vendors, as well as unauthorized use of the VIPPS seal and online drug seller marketing of shortage vaccines as over-the-counter drugs [31].

In addition, drug shortages, documented and publicized safety risks, and sales profiteering by gray market wholesalers not only may lead patients to attempt to procure shortage drugs through the Internet, but also may lead some providers to seek out shortage drug treatments from these online vendors. Not only is online purchasing from these vendors inherently dangerous, with compromised and counterfeit medications injuring and killing unsuspecting patients globally [13], but also scarcity in the legitimate market questions the very premise that these online drug sellers are selling authentic medication. Given current market-based demands and shortages, it does not appear that online drug sellers would have such an abundant supply of shortage drugs as advertised, and health care providers should regard these offers as highly suspect.

Indeed, even if these shortage drugs were authentic, Not Recommended online drug sellers, particularly international-based sites highly prevalent in this study, would be unlikely to have adequate knowledge and impetus to ensure proper transport [8]. Many of these drugs, including biologics, are sensitive and require special temperature controls and handling to retain therapeutic effectiveness—quality concerns that may not be known or of concern to suspect vendors [10,31]. Previous reports have also noted issues with online drug sellers’ storage and transport of sensitive drugs [33,34].

The dominance of international online sellers should be of particular concern, as the FDA does not permit personal drug importation from international sites due to its lack of ability to control quality and oversight of foreign materials [35]. Importing these drugs through purchase from these online drug sellers consequently violates the US Food, Drug, and Cosmetic Act [35]. Further, an argument for importation under an FDA personal importation exemption is inapplicable, even for the vast majority of these drugs, since it generally requires both that the drug be commercially unavailable in the United States and that use of any approved importation be medically supervised [36].

Moreover, it should also be noted that, from the patient’s perspective, drugs purchased from nondomestic sources are not eligible for public program reimbursement. Such purchases may lead to unnecessary patient expenditures, including remediation expenditures for additional care [36].

 This online environment with its limited regulation and cloaked nature as reviewed by Orizio et al [37], combined with the current drug shortage, is placing tremendous strain on the safety of the drug supply and fueling illicit activity. This situation exploits the desperate nature of vulnerable patients seeking any treatments that could potentially influence the course of their disease, similar to the Laetrile frauds for cancer treatments from the 1970s [26,38]. With the addition of reports that hospitals are increasingly sourcing drugs in shortage from the gray market [8,9], providers and patients seeking these therapies are surrounded by safety threats.

In response, at a minimum, risk management approaches should be considered. The US Customs and Border Protection agency is overwhelmed in attempting to monitor incoming drugs. It cannot destroy contraband suspect drugs coming through the mails due to international postal conventions. In general, it is limited to returning the package and contents to the original sender (for potential illicit resale) if not assessed within 24 hours, or simply allowing it to be sent on for delivery[14,39-41]. Hence, demand-side actions should be a focus.

Providers seeking drugs in short supply should verify the authenticity of the drug pedigree (ie, its documentation of transport) and should take advantage of professional resources for safe sourcing of drugs [10,42]. This includes implementing facility safety and regulatory protocols ensuring drug sourcing through accredited wholesalers and vendors that can and do certify that they procure drugs from legitimate, accredited distributors in compliance with pharmaceutical distributor laws, licensure, and regulations. NABP also provides accreditation here, and providers should purchase only from these NABP-accredited vendors [43].

This approach of listing and updating legitimate wholesale sources of drugs by a neutral, trusted entity should be considered as a strategy more broadly to allow rapid determination of vendor legitimacy on regional levels globally. These regional entities can then share information as to identified problematic vendors for information coordination, as well as determining and communicating best practices in promoting safety measures in the drug supply chain.

Patients should also be counseled about the dangers of counterfeit and substandard drugs when purchasing drugs online, and be advised to purchase only from verified NABP VIPPS-accredited online drug sellers as recommended by the FDA [16]. They should also be encouraged to inquire about provider drug-procurement policies, and require that drugs administered to them have appropriate pedigree evidencing authenticity. The patient is the last barrier to harm and should be partnered with and actively engaged in patient safety efforts. Such an approach is amenable to application in other health-delivery environments as well, with consistent public health messages and patient education such as that promulgated by important global health groups such as the International Council of Nurses [44]. These public health messages should expressly note global drug regulatory authority warnings and updated information on the dangers of purchasing drugs—including shortage drugs—online [18,19].

 These drug shortages have historically been a problem in clinical care, and have been the subject of past calls for reform and attention due to their adverse impact on public health [10,45-48]. Indeed, drug shortages have tripled in the last 6 years, while few have left the list, portraying a worrying trend [3], while the global nature of the problem has become increasingly dire [49,50].

Beyond reactive approaches, organized, proactive planning by stakeholders to address this issue should be started as soon as possible. Existing calls for enhanced drug regulatory authority powers to address drug shortages, stronger regulation of pharmaceutical distribution and pricing incentives, and more stringent penalties against illegal profiteering activities should be supported by stakeholders’ joining together in public–private partnership models to plan for new challenges in pharmaceutical supplies and safety.

These public–private partnerships should include multisector engagement with pharmaceutical manufacturers (including both brand and generic industries), drug regulators, patient safety and advocacy groups, hospitals, group purchasing organizations, and professional societies to prioritize and develop organized responses to drug shortages. Domestic and regional best practices can be applied in proactive efforts as well, with shared systems planning and strategies among stakeholders integrated across geopolitical and health-delivery environments. At the outset, a risk management approach may be best for priority planning—for example, public–private partnerships organized around supplying chemotherapy drugs and emergency department treatments, whose absence may have the greatest potential adverse clinical impact. In addition, due to the security threat illegal pharmaceutical sales pose and the need to address drug shortages as a national emergency, national security agencies and legal authorities should also be included in these public–private partnerships.

Integrated industry, regulator, patient, and provider partnerships would be able to better anticipate potential shortages and their impact on patient safety, and to help develop information resources and responses for stakeholders that are currently lacking for these situations [51]. Further, these partnerships would enhance existing calls for more advanced notification of shortages by manufacturers through establishing lines of communication [3,47,52,53] and would help to develop market-based incentives to encourage investment in manufacturing of shortage drugs, such as reimbursement-rate adjustment and creative financing for increased production [6,54].

Finally, strategic plans for safe substitutions of shortage drugs that could be integrated into emergency planning for regional delivery systems, along with voluntary international coordination of emergency supplies, should be considered in particularly needed areas and populations (such as public health treatments for pandemics or other communicable disease events). These partnerships would be similar to local coalition efforts to reallocate drugs subject to shortages by borrowing, back-ordering, increasing surveillance and legal enforcement against potential counterfeits, and seeking safe alternative supply sources through greater regional coordination [8,33].

 We note that this study has several limitations. It evaluates the drugs on the FDA Current Drug Shortages list as of one point in time, and its assessment of online availability similarly is limited to that period. Further, websites arise, change, and are taken down dynamically on the Internet, so the information and websites found in this study are necessarily limited as well. NABP VIPPS-accredited online pharmacies and NABP Not Recommended site designations also are limited in validity over time due to changes in the Internet and vendors. Finally, we did not determine the actual quality of products through test purchasing, as buying drugs for a fictional patient raises ethical and legal concerns.

 Overall, drugs on the FDA Current Drug Shortages list are critical therapeutic tools in the medical arsenal. Because of the crisis of access to these drugs, sources such as the Internet may be considered convenient for procurement. However, suspect vendors appear to dominate online marketing of shortage drugs. This high-risk digital conduit of medicines should be addressed. Reactive and proactive risk management strategies should be engaged to ensure that providers render optimal, safe care to patients, and that patients who are considering purchasing online due to desperate circumstances do not become the next victims of the drug shortage. Extreme caution and caveat emptor may best describe the themes when approaching this eHealth market.

## Abbreviations

FDA: Food and Drug Administration
NABP: National Association of Boards of Pharmacy
PPDL: personal page data link
